# Novel drug target identification for the treatment of dementia using multi-relational association mining

**DOI:** 10.1038/srep11104

**Published:** 2015-07-08

**Authors:** Thanh-Phuong Nguyen, Corrado Priami, Laura Caberlotto

**Affiliations:** 1 The Microsoft Research, University of Trento Centre for Computational Systems Biology (COSBI), Piazza Manifattura 1, 38068, Rovereto, Italy; 2 Life Sciences Research Unit, University of Luxembourg, 162 A, avenue de la Faïencerie, L-1511 Luxembourg; 3 Department of Mathematics, University of Trento, Via Sommarive, 14-38123 Povo, Italy

## Abstract

Dementia is a neurodegenerative condition of the brain in which there is a progressive and permanent loss of cognitive and mental performance. Despite the fact that the number of people with dementia worldwide is steadily increasing and regardless of the advances in the molecular characterization of the disease, current medical treatments for dementia are purely symptomatic and hardly effective. We present a novel multi-relational association mining method that integrates the huge amount of scientific data accumulated in recent years to predict potential novel targets for innovative therapeutic treatment of dementia. Owing to the ability of processing large volumes of heterogeneous data, our method achieves a high performance and predicts numerous drug targets including several serine threonine kinase and a G-protein coupled receptor. The predicted drug targets are mainly functionally related to metabolism, cell surface receptor signaling pathways, immune response, apoptosis, and long-term memory. Among the highly represented kinase family and among the G-protein coupled receptors, DLG4 (PSD-95), and the bradikynin receptor 2 are highlighted also for their proposed role in memory and cognition, as described in previous studies. These novel putative targets hold promises for the development of novel therapeutic approaches for the treatment of dementia.

Neurodegenerative dementia (ND) is a multi-faceted cognitive impairment that is progressive and irreversible due to deterioration of brain cells and their interconnections. It involves multiple cognitive deficits manifested by memory impairment and cognitive disturbances. The understanding of the genetic basis of ND has advanced in recent years, giving some insights into disease pathophysiology, but there are still major knowledge gaps in understanding the molecular mechanism underlying dementia. Dementia can be caused by a wide variety of diseases including more frequent pathologies such as Alzheimer’s disease, but also rare ones including Pick’s disease. Despite the high prevalence of dementia in the population, no drug treatments are available that can provide a cure. The two main classes of drugs available to treat Alzheimer’s disease, cholinesterase inhibitors and NMDA receptor antagonists, can only ameliorate the symptoms, or temporarily slow down the disease progression^1^, but they are not efficacious in treating the disease. Thus, due to the constant and rapid increase of life expectancy with an epidemic progression of neurodegenerative disorders, particularly Alzheimer’s disease^2^, it becomes very urgent to understand the molecular basis of dementia and to develop novel efficacious treatments.

The identification of novel drug targets (DTs) is of great importance for the development of new pharmaceutical products^3^, but the traditional drug discovery process is often laborious and expensive^4^. Systems biology can contribute to this field of research through an integrated view, capturing the complexity of the systems and integrating the huge amount of scientific data accumulated and archived in recent years. In such a situation, computational methods have become more and more essential to mine high-throughput data and discover useful knowledge for drug discovery in general and drug target identification in particular^3^,^5^,^6^,^7^,^8^,^9^. Among a wide range of approaches, the molecular network-based approach has the potential for the identification of DTs^8^,^10^. Molecular networks are very informative in studying human diseases and drugs because it is well-known that most molecular components do not perform their biological function in isolation, but interact with other cellular components in an intricate interaction network^11^,^12^,^13^. Emig *et al.* employed the network propagation and random walk method to predict DTs^14^. The domain-tuned-hybrid method was proposed to infer the network of drug-target interactions^15^. By analyzing human protein-protein interaction network, Milenković *et al.* developed a graphlet-based measure of network topology to predict potential drug targets^16^. Although previous works have been paving the way to the prediction of DTs, there exists a limiting factor in such data-intensive work due to the use of a single data source. Instead, it is essential to integrate the rich sources of *-omic* data (from the molecular to the network level) to acquire a comprehensive coverage of biomedical properties relevant to drug discovery.

In this study, we present a novel integrative approach to predict potential new drug targets for dementia based on multi-relational association mining (MRAM), an advanced data mining technique able to manipulate heterogeneous data without any information loss. The diseases studied are: Frontotemporal dementia (FTD), Alzheimer disease (AD), Lewy bodies disease (LBD), Progressive supranuclear palsy (PSP), Corticobasal dementia (CBD), Pick’s disease, Prion disease, Huntington’s disease, and Amyotrophic lateral sclerosis-Parkinsonism/dementia complex. The investigation was based on the list of known dementia DTs curated in^17^ with the integration of protein interaction network (PIN) and biological data from the Reactome, Gene Ontology, and InterPro databases. MRAM combined multiple relational data and achieved a better computational performance than other data mining techniques. Our method was able to predict novel DTs by inferring predictive association rules that were used to run testing experiments on the set of putative DTs that have direct interactions with both dementia-related genes and dementia DTs in the PIN described in^17^.

Our systems biology approach identified a series of potential novel DTs functionally associated to metabolism, cell surface receptor signaling pathways, immune response, apoptosis, and long-term memory. Among the predicted DTs, numerous serine threonine kinases, such as DLG4 (PSD-95) and a G-protein coupled receptor, Bradykinin receptors 2, were highlighted which could be considered for the development of innovative therapeutic approaches for the treatment of dementia.

## Materials and Methods

**Figure 1:**
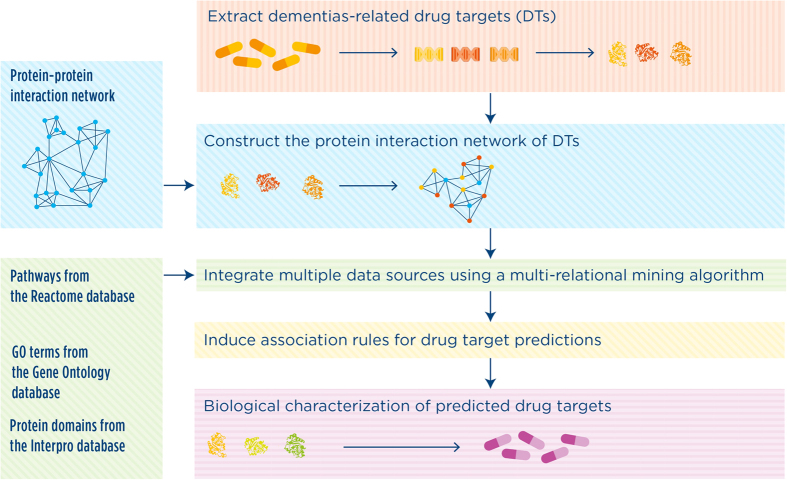
The systematic workflow of our methodological approach. Drug targets (DTs) were obtained by collecting information from different pharmaceutical company websites in the different phases of the drug discovery process (in red, yellow and orange). The interaction network of DTs was then constructed by extracting the direct 1-step neighbors of the DT based on the i2d database (the blue nodes in the network). Following the integration of multiple and heterogeneous data types by using the MRAM method, the rules were induced to predict the potential DTs. Finally, We characterized the functionality of the potential DTs by testing over-represented Gene Ontology biological process terms and pathways.

The pipeline that we applied is presented in Fig. 1 and consists of five steps as follows.

1. Extraction of molecular targets of drugs in different phases of the drug discovery process (from preclinical to marketed drugs);
2. Construction of a protein interaction network including the 1-step neighbors of DTs;
3. Integration of heterogeneous data from multiple databases (listed in Table 1);
4. Induction of association rules for DT prediction by using the MRAM algorithm;
5. Biological interpretation of the predicted DTs.

### Curation of dementia-related drug targets

Drug molecular targets were obtained by collecting information from different pharmaceutical company websites, from a clinical trial database (www.clinicaltrials.gov) and from the DrugBank database^18^. Drugs for the treatment of dementia in all phases of the drug discovery process, from preclinical to marketed drugs, were included. Although this approach is considering targets with lower (drugs in preclinical phases) and higher (marketed drug) level of confidence, it allowed obtaining the broadest coverage of the genes of interest for pharmaceutical drug development to identify the overall key molecular targets of interest for the treatment of dementia. We did not consider the overall pharmacological activity of the compounds, but only the primary targets of the drugs. From the set of DTs, we converted gene symbols to UniProt protein accessions using the identifier mapping scheme provided by the UniProt database^19^, obtaining the set of 268 DT proteins reported in Supplementary Material S1.

### Construction of the interaction network of drug targets

PINs are becoming increasingly comprehensive and they provide a better way for the understanding of the interaction among molecules than gene networks^11^,^20^. Our PIN was obtained from the Interologous Interaction Database (i2d)^21^. The i2d database stores two types of interactions: the *source interactions* curated from the majority of well-known data sources such as HRPD, BIND, BioGrid, DIP, IntAct, and the *predicted interactions* obtained by a homology-based approach. To increase the reliability of the protein interaction data we only considered the 183,524 source interactions *homo sapiens*-related.

**Figure 2:**
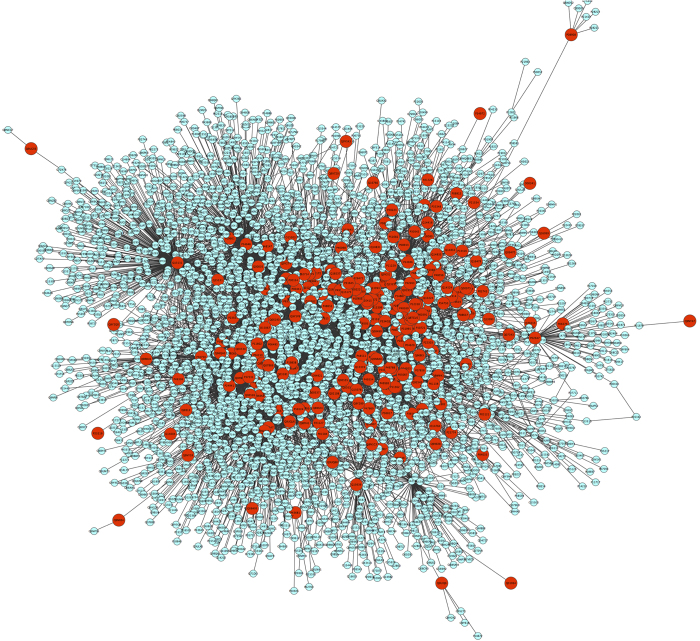
Interaction network of drug targets including drug targets and their first neighbors as extracted from the i2d database. The DTs are highlighted in red.

Based on the set of mapped DTs, we extracted the PIN by processing raw data of protein-protein interactions (PPI) in the i2d database. The final PIN of interest contained the DTs (nodes) and their direct interactions (edges). In this study, we considered one-step neighbors. The network was undirected and unweighted because we considered the binary interactions. Figure 2 illustrates the resulting PIN of dementia DTs. Protein identifiers are the UniProt ID accession number and gene identifiers are represented by the official gene symbols.

### Combination of heterogeneous data from multiple data sources

We considered both topological relationships between the DTs and their network neighbors and functional data representing biological properties of the DTs.

Regarding the topological data features, we calculated the number of a protein’s neighbors in our PIN, i.e. the degree centrality index of proteins formally defined as the cardinality of the set DC(p_*i*_) = {*p*_*j*_ ∈ *N* |*e*_*ij*_ ∈ *E*}, where *e*_*ij*_ denotes an interaction connecting *p*_*i*_ and *p*_*j*_, and *E* is the set of interactions. Degree centrality is one of the main measures used to study hubs of a network. We also considered articulation proteins. A protein is an articulation point in a network *iff* removing it (and the interactions through it) disconnects the network. These topological properties help elucidating the role of the DTs in the PIN.

Regarding the functional data features, we investigated three different kinds of properties: GO term, biological pathway and protein domain. The GO terms in the Gene Ontology database^22^ are divided into three categories: molecular function, biological process, and cellular component, and this information is used for the DT prediction. Since DTs most likely are part of the same cellular pathways, data extracted from the Reactome pathway database^23^ were analyzed. Protein domains are defined as structural or functional elements within a protein and affect the way that one protein interacts with one another. The protein domains of the DTs were obtained from the InterPro database^24^.

A multi-relational scheme was structured in form of tables and relationships between tables in the SQL Server Database Management to store our knowledge base as described above. The multi-relational scheme shows its advantage in the data integration because the data types are heterogeneous: GO terms, pathways, and protein domains are categorical free text while the degree centrality indexes are numerical and the articulation point feature is boolean.

### Prediction of drug targets using multi-relational association mining

#### MRAM and its applications

Most of the existing data mining algorithms seek data patterns in single tables. However, many datasets are inherently multi-relational and the information systems that manage them rely on multi-relational databases (MRDs). Multi-relational data mining (MRDM) open the way for handling and mining data in multiple tables (relations) directly in a MRD^25^,^26^,^27^. In MRDM, data are represented in a relational form where the records of the target table are potentially related to several records in secondary tables in one-to-many or many-to-many relationships. Three popular MRDM techniques are classification, clustering, and association. Association techniques (called multi-relational association mining - MRAM) have been successfully applied in bioinformatics, for example the analysis of gene set enrichment^28^, the prediction of hepatitis patients^29^, the analysis of different types of cancers based on microarray data^30^, the detection of potential adverse drug reactions^31^, and the prediction of protein interactions^32^. MRAM mines the data directly in their original structure of multiple relational tables, not requiring any pre-processing stage to generate a single table as in classical association mining (AM) algorithms like Apriori^33^ and FP-growth^34^. We developed an MRAM approach to exploring multiple data from a wide range of data sources to predict the dementia DTs.

#### Predicting drug targets using MRAM

**Figure 3:**
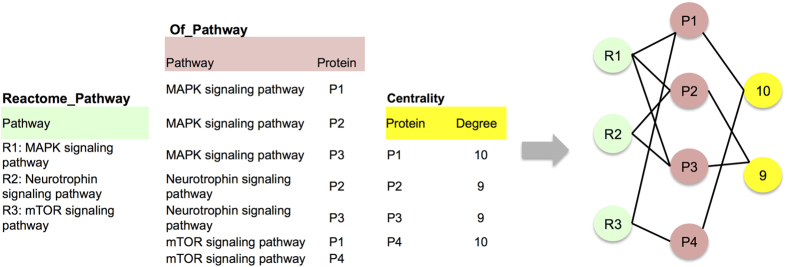
Example of a MRD in table form (left) and in graph form (right). The entity types ‘pathway’, ‘protein’, and ‘degree’ correspond to different blocks in the graph and the entities of each type correspond to different nodes. The table ‘Reactome_Pathway’ defines the pathway description. The join table ‘Of_Pathway’ defines a many-to-many relationship between the entity types ‘pathway’ and ‘protein’ and the table ‘Centrality’ defines an one-to-many relationship between entities ‘protein’ and ‘degree’. Two entities are linked with an edge if they co-occur in a same tuple.

The extracted data from GO, i2d, InterPro and Reactome are represented as relational tables in the Microsoft SQL server management system. Later one-to-many or many-to-many relationships among the tables were established. For example, many proteins may have the same degree centrality (one-to-many relationship) and on the other side a protein may belong to many Reactome pathways and one Reactome pathway has many proteins involved (many-to-many relationship). Figure 3 illustrates an example of extracted data in a multi-relational table form where ‘pathway’, ‘protein’, and ‘degree’ are three entity types, shown at the left-hand side of the figure. There is a relationship type between ‘pathway’ and ‘protein’, specifying the pathways that the proteins take part in, and between ‘protein’ and ‘degree’, specifying the degree centrality corresponding to a protein. The first relationship type is a many-to-many while the second is one- to-many. Note that the heterogeneity in the data makes the use of classical AM unsuitable in mining multiple relational data and underlines the importance of using an MRAM algorithm for the identification of the DTs.

The MRAM algorithm to explore data in multiple relational tables was employed by using the SQL Server 2012 Analysis Services (SSAS). The MRAM algorithm in the SSAS package uses optimization techniques to save space and make processing faster. Similar to traditional AM, MRAM handles data as items and group of items, called itemset. An association model consists of a series of itemsets and rules of the form *X ⇒ Y*, where *X* and *Y* are disjoint itemsets *X* ∩ *Y* = ∅. The algorithm finds rules within a dataset based on two parameters, *support* and *probability/confidence*. *Support* is the occurrence frequency of the targeted item or itemset in a given dataset. *Probability* is the co-occurrence frequency of items in *Y* and *X.*

The inputs of the algorithm are *(1)* positive training examples as the set of known DTs, denoted *S*^*pos*^*, (2)* negative training examples as the set of non-DT selected randomly from the set of proteins which do not belong to *S*^*pos*^, denoted *S*^*neg*^, and *(3)* a five-dimension vector representing degree centrality *f*_*deg*_, articulation point *f*_*art*_, GO term *f*_*GO*_, Reactome pathway *f*_*path*_, and protein domain *f*_*dom*_. The MRAM algorithm traverses the input dataset in multiple tables to find items that appear together in a case. The algorithm then groups into itemsets any associated items that have *support* greater than a threshold MINIMUM_SUPPORT. *Probability* is calculated for each rule and the algorithm restricts the number of rules based on a threshold parameter MINIMUM_ PROBABILITY. The resulting rules were used to infer new putative DTs.

### Gene ontology and pathway analysis

We run the proposed method to predict putative DTs from the set of connector genes identified in our previous work^17^. Connector genes being directly linked to both the dementia disease genes extracted from the OMIM database^35^ and the DTs extracted in this study are likely to have more chance to be relevant for the disease and, thus, for being potential DTs.

The newly predicted DTs were used to extract the most representative GO biological process terms (i.e., the ones that are over-represented, but that do not refer to most general biological processes). For identifying and visualizing enriched GO terms, we used GOrilla^36^ and REVIGO web-based tools^37^. Hypergeometric distribution was applied to test GO term enrichment, and a p-value threshold of 0.05 was selected. Pathway enrichment analysis and disease association was performed using DAVID web-based tool^38^.

## Results

221 DTs out of 268 known DTs have at least one interacting neighbor in our PIN. Therefore the positive example *S*^*pos*^ consists of 221 DTs. Since there are no available negative examples (non-DTs), we randomly selected three sets of negative examples with different sizes (221, 500, and 1,000) to build the set *S*^*neg*^. The approach applied to different size examples proved to be scalable and stable. We obtained a network of 3,112 proteins and 6,541 interactions that is well connected with the average shortest path length equal to 3 and the average number of neighbors equal to 4. We obtained 44,433 GO terms, 11,738 InterPro domains, and 4,240 Reactome pathways related to the 3,122 proteins.

**Figure 4:**
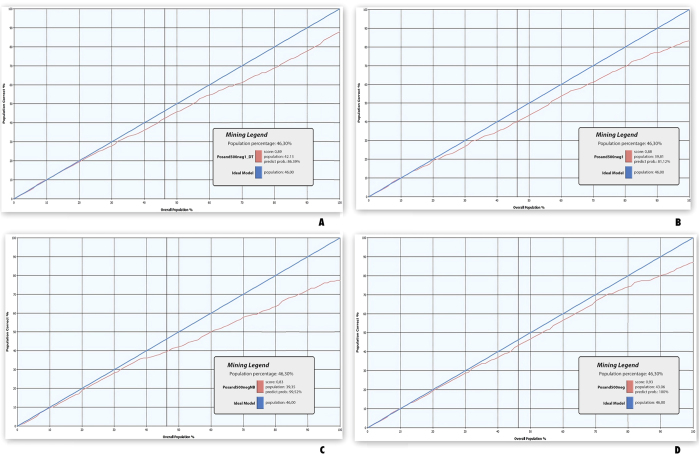
Computational performance of the multi-relational association mining (MRAM) method compared to the other methods. Fig 4A–D shows the lift charts of the Decision Tree method (D-Tree), the Naïve Bayes (NB) and, the Neural Network (NN), and (MRAM, respectively. The x-axis of the chart represents the percentage of the test dataset that is used to compare the predictions. The y-axis now represents the percentage of predictions that are correct. The blue lines show the performance of the ideal model and the red lines show the performance of D-Tree, the NB, NN, and MRAM models correspondingly.

To evaluate the performance of our method, we computed the lift charts for MRAM, Decision Tree (D-Tree), Naïve Bayes (NB), and Neural Network (NN) methods. These charts show how the model performs for all states of the predictable attribute. Figure 4A–D shows the lift charts of D-Tree, NB, NN, and MRAM with 500 negative examples. The lift chart of MRAM is closer to the ideal model than the other three methods. MRAM also achieved a better accuracy (93%) than D-Tree, NB, and NN that achieved 89%, 83%, and 88%, respectively. We then performed the 10-fold cross validation to compute Likelihood Log Score, Likelihood Lift, Likelihood Root Mean Square Error (RMSE)^39^, and area under the curve (AUC)^40^. Recall that the higher AUC, Likelihood Log Score, Likelihood Lift and the lower RMSE, the better performance. The experiments were performed for the four methods on the same set of data. Table 2 presents the average values calculated for 10 experiments corresponding to the 10-fold cross validation of the above-mentioned measures and the standard deviations calculated for the methods with the three sets *S*^*neg*^ of 221, 500 and 1,000 negatives. In all experiments, MRAM performed better than the other methods. To evaluate the contribution of each data feature, we did several experiments by excluding the features one-by-one and then computing the likelihood lift. Table 3 shows that the experiment with all data combined achieved the best result, and the next was the one excluding InterPro domain feature. The worst likelihood lift was obtained when excluding topological features. As a result, the topological feature contributes most and the InterPro domain feature contributes least to the method.

**Figure 5:**
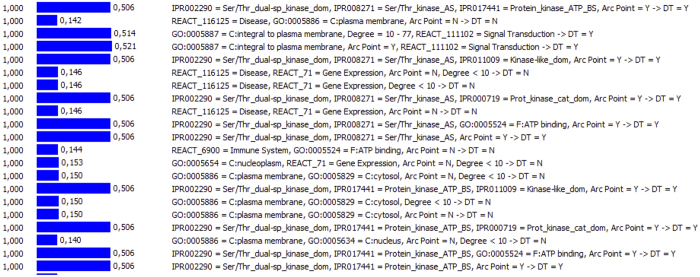
Representation of obtained association rules. Three columns: Probability, Importance, and Rule. The *probability* describes how likely the result of a rule is to occur. The *importance* measures the significance of a rule.

Figure 5 shows some of the induced rules with *probability* equal to 1 in three columns: Probability, Importance, and Rule. For example, the rule *“GO:0005887 = C:integral to plasma membrane, Degree *=* 10–77, REACT_111102 = Signal Transduction ⇒DT = Y”* has *probability* = 1 and *importance* = 0.514. The rule shows that one protein has chance to be a DT if it is integral to the plasma membrane, is central with at least 10 interactions (up to 77 interacting proteins), and takes part in the signal transduction pathway. The *probability* describes how likely the result of a rule is to occur. The *importance* measures the significance of a rule. The *importance* of a rule is calculated by the log likelihood of the right-hand side of the rule, given the left-hand side of the rule. For example, in the rule A *==> B*, MRAM calculates the ratio of cases with A and B over cases with B but without A, and then normalizes that ratio by using a logarithmic scale. Indeed a rule with high *probability* might be too general to provide useful information. The greater *importance,* the more significant the rule is. The top rules with *probability* = 1 and *importance* > 0.5 are presented in Supplementary File Table S2.

Functional enrichment analyses of GO biological process terms was performed for the list of predicted DTs, showing metabolic-related terms (regulation of glucose transport and of insulin signaling), cell surface receptor signaling pathways (Wnt, neurotrophin, MAPK cascade, and tachykinin receptor signaling), immune response-related terms (innate immune response and toll-like receptor singalling), apoptosis, and long-term memory (Fig. 5; Supplementary File Table S3).

Pathway analysis revealed similar results as the GO, but in addition indicates Alzheimer disease-amyloid secretase pathway, type 2 Diabetes, and metabotropic glutamate receptor group I pathways as associated with the predicted DTs (Supplementary File Table S4).

## Discussion

In recent years, wealth of information has been produced on neurodegenerative dementia and particularly on Alzheimer’s disease, and the integration of these data to obtain novel knowledge is one of the big challenges in modern neurobiology. This investigation employed a data mining method on heterogeneous data including categorical free text (i.e. GO terms and pathways), numerical values (i.e. the degree centrality and number of domain-domain interactions), and boolean values (i.e. articulation protein). The data were managed in a multiple relational database for which classical AM methods do not provide a suitable platform. MRAM is the most recent approach which aims to overcome the difficulties in multi-relational data integration. It enables direct pattern extraction from multiple relations, without the necessity of transferring data to a single relation^25^,^28^, thus avoiding computationally expensive joining operations and semantic losses caused by the representation limit of a single table with repetitions of many attributes and data. Because this merged table is large and sparse, the mining process becomes more expensive and time-consuming^41^. The experiments on the DT prediction showed that MRAM was the best among other well-known data mining techniques: DT, NB, and NN methods. With the rapid growth of public biological databases, MRAM can be widely applied to discover complex patterns through the rich relational structure and the mixed-up types of data.

**Figure 6:**
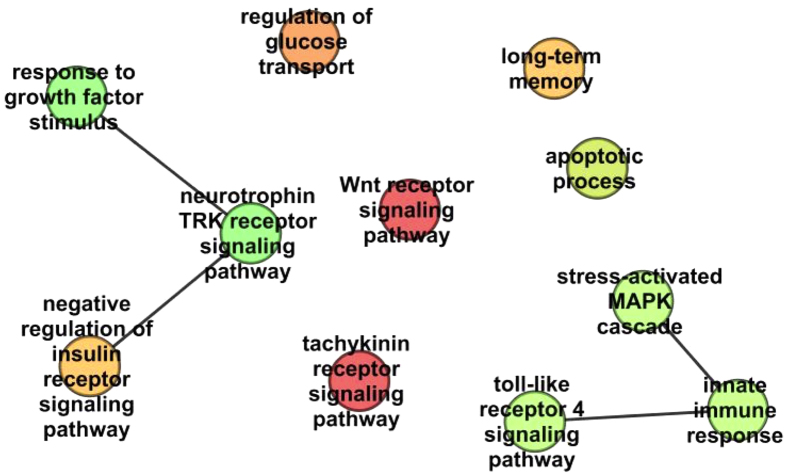
Summary of statistically significant Gene Ontology biological processes functional annotation corresponding to the putative DT list as obtained from REVIGO. Nodes are GO terms and edges represent the strongest GO terms pairwise similarity. Colors represent the p-values (low values in green, high in red). Only significant GO terms are shown (P < 0.001).

A significant enrichment in Alzheimer disease-amyloid secretase pathway (Panther:P00003), the hallmark of Alzheimer’s disease^42^ was evidenced by pathway analysis of the predicted DTs giving further support to the relevance of our findings to dementia. In addition, a significant enrichment in biological functions associated to long-term potentiation (Fig. 6), a phenomenon related to synaptic plasticity, one of the most important cellular mechanisms that underlies learning and memory, was also found. Proteins involved are PKC, PKA, ERK1/2, Rsk, and CAMK2 (Supplementary File 3). These results are in line with recent studies suggesting that activation of protein kinase C could have potential for the treatment of dementia^43^.

Interestingly, an association with type 2 diabetes pathway (KEGG:hsa04930) was also evidenced. Type 2 Diabetes is a major risk factor for Alzheimer’s disease and dementia and the concept that Alzheimer’s is fundamentally a metabolic disease that results in progressive impairment in the brain’s capacity to utilize glucose and respond to insulin/insulin like growth factor stimulation has recently gained increasing support^44^,^45^. Thus, in line with ours^17^,^46^ and other groups’^47^,^48^ previous findings, these results further emphasize the strong link between Alzheimer’s disease and, more generally, neurodegenerative dementia, to metabolic disorders and diabetes. This finding may suggest that a dementia drug could be used as treatment platforms for both diseases and their co-morbidities in view of the overlapping molecular pathway.

**Figure 7:**
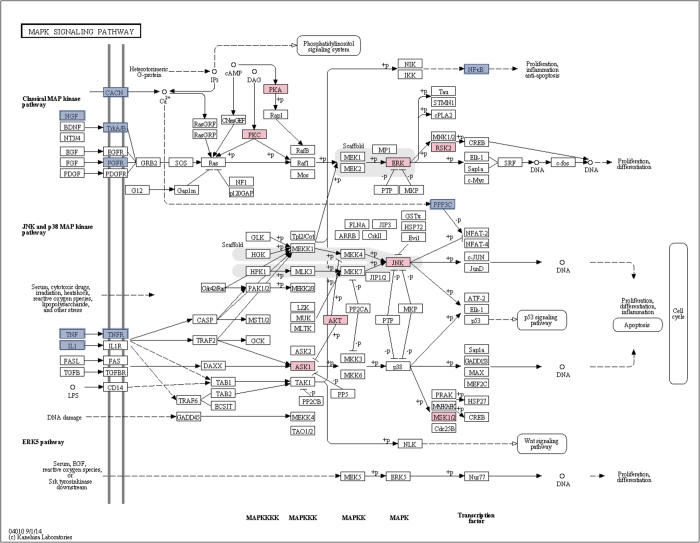
Flow diagram representing the molecular interactions in the MAPK signaling pathway (from KEGG database hsa04010)^59^. The pathway is enriched with predicted drug targets proteins, labeled in pink. In blue are labeled the drug targets for dementia.

Considering the most enriched GO terms and pathway, a major role for MAPK (KEGG: hsa04010) is evident (Figs. 6 and 7). The putative role of p38 MAPK as a new Alzheimer’s disease treatment strategy has emerged in recent years. p38 MAPK operates not only in response to stress and inflammatory reactions, but also in other events related to AD, such as excitotoxicity, synaptic plasticity, and tau phosphorylation^49^.

Finally, considering the degree index (Table 4), DLG4 (Discs, Large Homolog 4 or PSD-95), a postsynaptic marker playing a basic role in synaptic transmission by anchoring NMDA receptors and interacting with nNOS^50^,^51^, was the protein with the highest degree centrality. Its link to dementia, particularly in cognitive performances is also supported by animal studies: mice lacking PSD-95 have severe spatial memory deficits^52^, while mice exposed to enriched environments with improved learning and memory, have elevated PSD-95^53^. Finally, DLG4 has been linked to a genetic form of dementia: familial Danish dementia^54^. A high affinity molecule acting on PSD-95 has been previously identified and could possibly be used for Alzheimer’s disease as also suggested by Bach and collaborators.^55^.

Most of the putative DTs are kinases, particularly serine threonine kinases. In drug development, achieving selective inhibition of specific protein kinases is challenging since most small-molecule kinase inhibitors interact with multiple members of the protein kinase family^56^. Thus, among the predicted DTs in the G protein-coupled receptor family, a possible protein of interest could be the bradikynin receptor 2 (BDKRB2). Bradikynin and related kinins are a family of small peptides which act as mediators of inflammation and pain and that transmit their biological effects via G protein-coupled receptors through the action on two bradykinin receptors, the B1 and the B2 subtypes^57^. Previous studies support the proposed effectiveness of an action on BDKRB2 for the treatment of dementia, demonstrating the ability of a BDKRB2 antagonist (HOE 140) in reversing the spatial learning and memory deficits induced by Aβ peptide in an animal model of Alzheimer’s disease^58^. Thus, further studies are needed in this direction to confirm the validity of this target for future development.

## Conclusions

Our systems biology approach was able to integrate previous existing knowledge in dementia to identify novel molecular targets for the development of innovative therapeutic intervention. A series of kinase including DLG4 (PSD-95) and BDKRB2, a G protein-coupled receptor of the kinin family were identified, but further studies are needed to confirm this finding and the druggability of the proposed targets.

## Additional Information

**How to cite this article**: Nguyen, T.-P. *et al.* Novel drug target identification for the treatment of dementia using multi-relational association mining. *Sci. Rep.* **5**, 11104; doi: 10.1038/srep11104 (2015).

## Supplementary Material

Supplementary Information

Supplementary Tables

## Figures and Tables

**Table 1:** Reference databases used for data retrieval during the investigation.

**Database**	**Description**	**URL**	**Statistics**	**Data extracted**
Clinical drug data	a database of publicly and privately supported clinical studies of human participants conducted around the world	www.clinicaltrials.gov		drug target
DrugBank	a bioinformatics and cheminformatics resource that combines detailed drug data with comprehensive drug target (i.e. sequence, structure, and pathway) information	http://www.drugbank.ca/	4,092 unique drug targets	drug target
i2d	an on-line database of known and predicted mammalian and eukaryotic protein-protein interactions	http://ophid.utoronto.ca/	183,524 curated interactions for human	protein interaction
Reactome	a curated resource of core pathways and reactions in human biology.	http://www.reactome.org	1,597 for human	pathway
InterPro	an integrated database of predictive protein "signatures" used for the classification and automatic annotation of proteins and genome	http://www.ebi.ac.uk/interpro/	7,497 protein domains	protein domain
Gene Ontology	a relational database comprised of the GO terms as well as the annotations of genes and gene products to terms in the those ontologies	http://geneontology.org/		GO term

**Table 2:** Computational measures calculated for the four methods with the three sets of negative examples with different sizes *n*_*1*_, *n*_*2*_, *n*_*3*_.

**Measure Method**	**AUC**	**Likelihood Log Score**	**Likelihood Lift**	**Likelihood RMSE**
*n*_*1*_ = 221				
MRAM	**0.846**	**−0.259 ± 0.021**	**0.433 ± 0.020**	**0.211 ± 0.001**
Decision Tree	0.837	−0.405 ± 0.063	0.287 ± 0.063	0.213 ± 0.020
Bayesian Network	0.822	−0.540 ± 0.175	0.152 ± 0.175	0.284 ± 0.029
Neural Network	0.783	−0.416 ± 0.084	0.276 ± 0.084	0.224 ± 0.026
*n*_*2*_ = 500				
MRAM	**0.890**	**−0.149 ± 0.010**	**0.469 ± 0.019**	**0.113 ± 0.016**
Decision Tree	0.827	−0.294 ± 0.102	0.314 ± 0.102	0.115 ± 0.009
Bayesian Network	0.814	−0.348 ± 0.073	0.276 ± 0.073	0.116 ± 0.031
Neural Network	0.783	−0.501 ± 0.108	0.114 ± 0.108	0.237 ± 0.018
*n*_*3*_ = 1,000				
MRAM	**0.883**	**−0.211 ± 0.056**	**0.256 ± 0.054**	**0.063 ± 0.007**
Decision Tree	0.866	−0.265 ± 0.039	0.202 ± 0.040	0.166 ± 0.001
Bayesian Network	0.808	−0.263 ± 0.048	0.218 ± 0.047	0.130 ± 0.025
Neural Network	0.804	−0.293 ± 0.060	0.198 ± 0.057	0.174 ± 0.019

The best results obtained are labeled in bold.

**Table 3:** Performance of MRAM in term of likelihood lift with different subsets of data features and the three sets of negative examples with different sizes *n*_*1*_, *n*_*2*_, *n*_*3*_.

**Experiment**	***n***_***1***_** = 221**	***n***_***2***_** = 500**	***n***_***3***_** = 1,000**
Exp1: All data features excluding the topological data features	0.391	0.412	0.211
Exp3: All data features excluding the GO data feature	0.398	0.443	0.220
Exp4: All data features excluding the Reactome data feature	0.408	0.436	0.215
Exp5: All data features excluding the InterPro data feature	0.411	0.449	0.237
Exp6: All of investigated data feature	**0.433**	**0.469**	**0.256**

The best results obtained are labeled in bold.

**Table 4:** List of predicted drug targets with UniProt ID, Official gene symbol, Gene name and Degree centrality.

**UniProt ID**	**Official Gene Symbol**	**Gene Name**	**Degree**
P78352	DLG4	discs, large homolog 4 (Drosophila)	18
P17612	PRKACA	protein kinase, cAMP-dependent, catalytic, alpha	17
P28482	MAPK1	mitogen-activated protein kinase 1	16
P05771	PRKCB	protein kinase C, beta	14
Q05655	PRKCD	protein kinase C, delta	13
P31749	AKT1	v-akt murine thymoma viral oncogene homolog 1	12
P68400	CSNK2A1	casein kinase 2, alpha 1 polypeptide pseudogene; casein kinase 2, alpha 1 polypeptide	11
P68400	CSNK2A1P	casein kinase 2, alpha 1 polypeptide pseudogene; casein kinase 2, alpha 1 polypeptide	11
Q96RR4	CAMK2A	calcium/calmodulin-dependent protein kinase kinase 2, beta	10
P27361	MAPK3	hypothetical LOC100271831; mitogen-activated protein kinase 3	9
Q8TD19	NEK9	NIMA (never in mitosis gene a)- related kinase 9	9
Q05513	PRKCZ	protein kinase C, zeta	7
Q9UQM7	CAMK2a	calcium/calmodulin-dependent protein kinase II alpha	7
P25098	ADRBK1	adrenergic, beta, receptor kinase 1	6
Q02156	PRKCE	protein kinase C, epsilon	6
O00141	SGK1	serum/glucocorticoid regulated kinase 1	5
P45983	MAPK8	mitogen-activated protein kinase 8	5
P51812	RPS6KA3	ribosomal protein S6 kinase, 90kDa, polypeptide 3	5
Q15831	STK11	serine/threonine kinase 11	5
Q96L34	MARK4	MAP/microtubule affinity-regulating kinase 4	5
Q9Y6E0	STK24	serine/threonine kinase 24 (STE20 homolog, yeast)	5
P19784	csnk2a2	casein kinase 2, alpha prime polypeptide	4
P23443	RPS6KB1	ribosomal protein S6 kinase, 70kDa, polypeptide 1	4
P34947	GRK5	G protein-coupled receptor kinase 5	4
O94985	CLSTN1	calsyntenin 1	3
P30411	BDKRB2	bradykinin receptor B2	3
P43250	GRK6	G protein-coupled receptor kinase 6	3
Q15418	RPS6KA1	ribosomal protein S6 kinase, 90kDa, polypeptide 1	3
Q16512	PKN1	protein kinase N1	3
Q16659	MAPK6	mitogen-activated protein kinase 6	3
O75582	RPS6KA5	ribosomal protein S6 kinase, 90kDa, polypeptide 5	2
Q99683	MAP3K5	mitogen-activated protein kinase kinase kinase 5	2
Q9NSB8	HOMER2	homer homolog 2 (Drosophila)	2
Q9NSC5	HOMER3	homer homolog 3 (Drosophila)	2
