# Supplementary material for: Novel drug target identification for the treatment of dementia using multi-relational association mining
Source: Sci Rep. 2015 Jul 8;5:11104. doi: 10.1038/srep11104 (PMC4495601; doi:10.1038/srep11104)
Supplement: Supplementary Information [file srep11104-s1.pdf]

Novel Drug Target Identification for the Treatment of Dementia Using Multi-Relational Association Mining

Thanh-Phuong Nguyen, Corrado Priami, Laura Caberlotto

Supplementary Information

File type: SupplementaryMaterials_ Nguyenetal.xls

Description: Consist of four worksheets:

- **Supplementary Table S1**: List of the investigated drug targets.
- **Supplementary Table S2**: List of top predictive rules with *probability* = 1 and *importance* > 0.5.
- **Supplementary Table S3**: Gene ontology biological functions enrichment analysis results of all predicted drug targets.
- **Supplementary Table S4**: Pathway enrichment analysis results of all predicted drug targets
